# Crystal structure, electrochemical and spectroscopic investigation of *mer*-tris­[2-(1*H*-imidazol-2-yl-κ*N* ^3^)pyrimidine-κ*N* ^1^]ruthenium(II) bis­(hexa­fluorido­phosphate) trihydrate

**DOI:** 10.1107/S2056989018007995

**Published:** 2018-06-05

**Authors:** Naheed Bibi, Renan Barrach Guerra, Luis Enrique Santa Cruz Huamaní, André Luiz Barboza Formiga

**Affiliations:** a Institute of Chemistry, University of Campinas – UNICAMP, PO Box 6154, 13083-970, Campinas, SP, Brazil

**Keywords:** crystal structure, homoleptic complex, heteroaryl-imidazole, ruthenium(II), meridional isomer

## Abstract

The crystal structure of the title compound, [Ru(C_7_H_6_N_4_)_3_](PF_6_)_2_·3H_2_O, a novel Ru^II^ complex with the bidentate ligand 2-(1*H*-imidazol-2-yl)pyrimidine, comprises a complex cation in the *meridional* form exclusively, with a distorted octa­hedral geometry about the ruthenium(II) cation. The Ru—N bonds involving imidazole N atoms are comparatively shorter than the Ru—N bonds from pyrimidine because of the stronger basicity of the imidazole moiety. The three-dimensional hydrogen-bonded network involves all species in the lattice with water mol­ecules inter­acting with both counter-ions and NH hydrogen atoms from the complex. The supra­molecular structure of the crystal also shows that two units of the complex bind strongly through a mutual N—H⋯N bond. The electronic absorption spectrum of the complex displays an asymmetric band at 421 nm, which might point to the presence of two metal-to-ligand charge-transfer (MLCT) bands. Electrochemical measurements show a quasi-reversible peak referring to the Ru^III^/Ru^II^ reduction at 0.87 V *versus* Ag/AgCl.

## Abstract 2

The first example of a homoleptic Ru^II^ complex with heteroaryl-imidazoles is reported in the meridional stereochemistry, exclusively. The supra­molecular hydrogen-bonded network reveals mutual N—H⋯N bonds between adjacent complexes.

## Chemical context   

Since the first preparation of the tris­(2,2-bi­pyridine) ruth­enium(II) complex by Burstall (1936 ▸), its inter­esting electrochemical and photochemical properties have stimulated the preparation and characterization of numerous analogous ruthenium(II) complexes (Le-Quang *et al.*, 2018 ▸; Dong *et al.*, 2018 ▸; Linares *et al.*, 2013 ▸). When asymmetric bidentate ligands are used to obtain homoleptic complexes, *facial* and *meridional* isomers can be obtained, depending on steric and electronic properties with important implications on chemical reactivity and spectroscopy (Metherell *et al.*, 2014 ▸). An inter­esting class of asymmetric ligands are hetero­aryl-imidazoles, since a combination of electron-rich and electron-poor rings can be used to tune the electronic properties of the final complexes (Ratier de Arruda *et al.*, 2017 ▸; Nakahata *et al.*, 2017 ▸).

In this context, we have devised a synthetic procedure to obtain exclusively the *meridional* isomer of the first reported homoleptic Ru^II^ complex with the bidentate 2-(1*H*-imidazol-2-yl)pyrimidine (impm) ligand containing imidazole (im) and pyrimidine (pm) rings in the same unit.
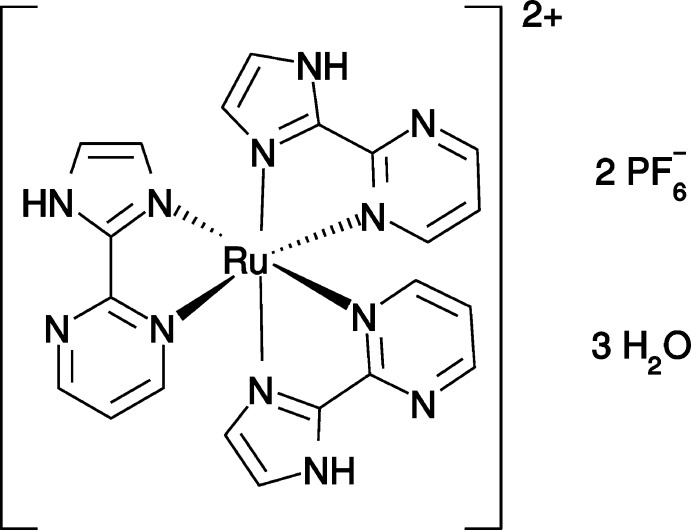


## Structural commentary   

**Figure 1:**
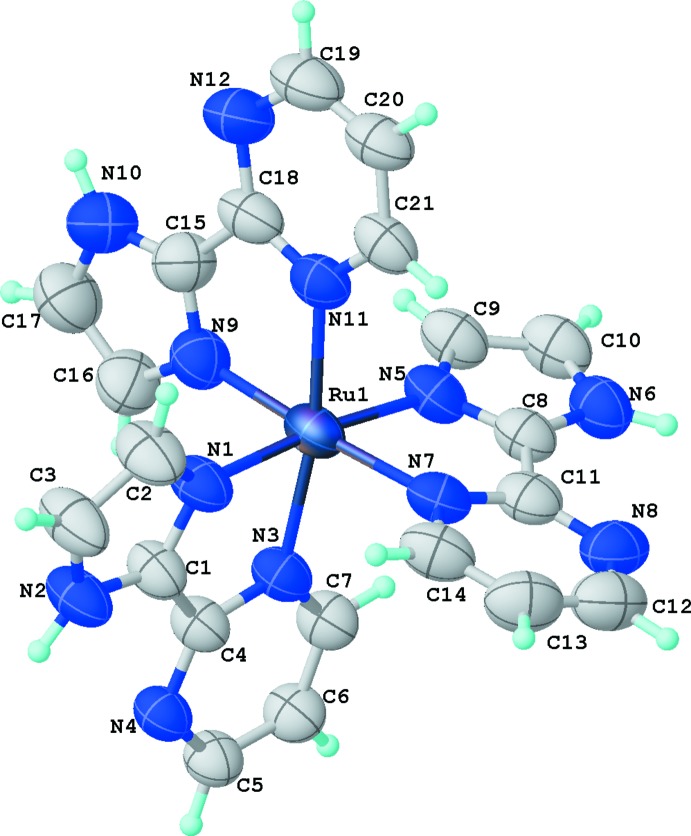
The mol­ecular structure of the homoleptic cationic complex [Ru(*L*)_3_]^2+^ (*L* = C_7_H_6_N_4_) with the atom-numbering scheme. Displacement ellipsoids are plotted at the 50% probability level.

The title complex crystallizes with two hexa­fluorido­phosphates counter-anions and three lattice water mol­ecules. The total +2 charge for the complex is in very good agreement with molar conductivity and mass spectrometry measurements. We can conclude that all three ligands in the complex are neutral, not showing the typical ionization of the imidazole hydrogen atom. The mol­ecular structure of the cationic complex is shown in Fig. 1 ▸. It reveals a distorted octa­hedral configuration with *meridional* stereochemistry, with two imidazole units *trans* to each other as well as two pyrimidine units *trans* to each other. There is no correlation between the *trans–cis* orientation and bond lengths. For example, all Ru—N_im_ bond lengths are essentially the same within their standard uncertainties, and the same observation is valid for Ru—N_pm_ bond lengths. However, Ru—N_im_ bond lengths are systematically shorter than Ru—N_pm_ bonds by 0.03 Å, as expected from the stronger Lewis basicity of the imidazole unit. Averaged bond lengths are 2.054 (10) Å for Ru—N_im_ and 2.083 (8) Å for Ru—N_pm_. As a result of the bidentate nature of the ligands, coordination angles differ from the ideal 90° value with N_im_—Ru—N_pm_ angles ranging from 78.5 (2) to 78.7 (2)°, the latter being the main cause for the distorted octa­hedral configuration.

## Supra­molecular features   

**Figure 2:**
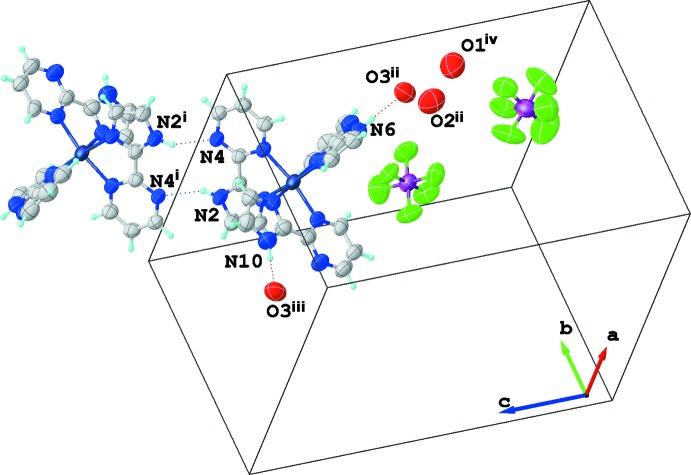
Outline of the unit cell with axes showing all mol­ecular entities in the crystal. Details of the hydrogen bonds found for the [Ru(*L*)_3_]^2+^ unit are also shown. Dashed lines indicate the mutual N—H⋯N array between two symmetric complexes through one of the heteroaryl-imidazole ligands and two hydrogen bonds with water mol­ecules. Symmetry codes: (i) −*x* + 1, −*y* + 2, −*z* + 2; (ii) *x* + 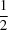, −*y* + 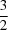, *z* − 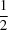; (iii) −*x* + 1, −*y* + 1, −*z* + 2; (iv) −*x* + 1, −*y* + 2, −*z* + 1. Water H atoms were not found, see text for details. Atoms of the PF_6_^−^ anion in the upper right corner have symmetry code (ii).

Although hydrogen atoms were not modelled for the three water mol­ecules present in the crystal structure, it is clear that a three-dimensional hydrogen-bonded network is formed by all species. Water mol­ecules cluster in triads and are close to two hexa­fluorido­phosphate anions in the lattice. The supra­molecular arrangement of water mol­ecules and PF_6_^−^ anions may result in different hydrogen-bonded patterns, and the disorder in hydrogen-atom positions may explain the absence of electron densities close to oxygen atoms in difference maps. Possible donor–acceptor pairs involving the water oxygen atoms are included in Table 1 ▸. One of the water mol­ecules (O3) is hydrogen bonded to two N—H imidazole units, N6 and N10, Fig. 2 ▸ and Table 1 ▸. A rather strong mutual inter­molecular inter­action between two [Ru(impm)_3_]^2+^ units through one of the ligands involving centrosymmetric N—H⋯N pairs completes the three-dimensional hydrogen-bonded network (Fig. 2 ▸).

## Electrochemistry and electronic spectroscopy   

The Ru^III^/Ru^II^ potential for the [Ru(impm)_3_]^2+^ complex (0.87 V *versus* Ag/AgCl) was found to be inter­mediate between those reported for [Ru(im)_6_]^2+^ (0.295 V; Clarke *et al.*, 1996 ▸), and [Ru(bpm)_3_]^2+^ (1.72 V; Ernst & Kaim, 1989 ▸), in which bpm stands for 2,2′-bi­pyrimidine. Since the reduction potential can be directly related to the *t*_2*g*_ orbitals of the complex, *i.e*. the HOMO (Possato *et al.*, 2017 ▸; Eberlin *et al.*, 2006 ▸; Nunes *et al.*, 2006 ▸), the changes in potential can be accounted for by the high imidazole electron σ-donor ability, which tends to increase the energy of the HOMO, leading to a decrease of the reduction potential. Conversely, pyrimidine is a better π-receptor, decreasing the HOMO energy, therefore increasing the reduction potential (Lever, 1990 ▸). The electrochemical results reveal that the impm ligand was successfully used to tune these effects by combining them, as we had intended. The electronic spectrum of [Ru(impm)_3_]^2+^ revealed an asymmetric band centered at 421 nm (log ∊ = 4.14), indicating that two superimposed metal-to-ligand charge-transfer (MLCT) bands may be present. This could be explained if two transitions from the Ru^II^ *t*_2*g*_ to two π* orbitals are observed. Moreover, the MLCT in [Ru(bpm)_3_]^2+^ is observed at 454 nm (Ernst & Kaim, 1989 ▸); this is an indication that the π* orbitals involved in the [Ru(impm)_3_]^2+^ transitions lie higher in energy.

## Database survey   

Surveys of the Cambridge Structural Database (CSD, Version 5.38, last update February 2018; Groom *et al.*, 2016 ▸) and *SciFinder* (SciFinder, 2018 ▸) revealed no hits. To the best of our knowledge, this is the first crystal structure reported for a homoleptic ruthenium complex with heteroaryl-imidazoles. The survey revealed the synthesis of the cationic complex [Ru(impy)_3_]^2+^ (Stupka *et al.*, 2005 ▸), in which impy is 2-(1*H*-imidazol-2-yl)pyridine, but no crystal structure was reported. However, we could relate to other similar crystals containing related cations [Ru(bpm)_3_]^2+^, [Ru(bpy)_3_]^2+^, or [Ru(bpz)_3_]^2+^, in which bpy is 2,2′-bi­pyridine and bpz is 2,2′-bi­pyrazine. [Ru(bpm)_3_]^2+^ contains a pyrimidine moiety with an Ru—N length of 2.067 (4) Å, similar to our complex, whereas [Ru(bpy)_3_]^2+^ and [Ru(bpz)_3_]^2+^ show Ru—N bond lengths of 2.056 (2) and 2.05 (1) Å, respectively (Rillema *et al.*, 1992 ▸). The only other complex in which impm appears as a ligand is with Cu^II^ and was reported by us (Nakahata *et al.*, 2017 ▸ *).* In the latter, similar to what we have observed in this work, the Cu—N_pm_ bond length is 2.078 (2) Å, which is a bit longer than that of Cu—N_im_ [1.975 (5) Å]. The mol­ecular structure of [Ru(im)_6_]^2+^ was found to have an average Ru—N length of 2.099 (2) Å (Baird *et al.*, 1998 ▸).

## Synthesis and crystallization   

The ligand was synthesized following the same procedure as reported in the literature (Nakahata *et al.*, 2017 ▸). The Ru^II^ complex was prepared by a mixture of one equivalent of RuCl_3_·3H_2_O (50 mg), 3.3 equivalents of the ligand (92 mg) and 10 ml of DMF. The mixture was stirred and heated to 423 K for 5 min, until the colour turned to green. After the addition of 45 µl of tri­ethyl­amine, the reaction mixture was kept under reflux for three h, resulting in a reddish purple mixture. This reaction mixture was filtered while still hot using a sintered glass funnel (G4). The filtrate was processed further with constant addition of ethanol and evaporation using a rotary evaporator until the volume reduced to almost 1.5 ml. The resulting reduced mixture was added dropwise to an aqueous solution of NH_4_PF_6_ (200 mg in 5 ml of milliQ water) and left in the refrigerator overnight to induce precipitation. Subsequently, the precipitate was filtered, washed with ice-cold water to remove excess NH_4_PF_6_ and dried in a desiccator. Yield: 83.42%. Analysis calculated for [Ru(C_7_H_6_N_4_)_3_](PF_6_)_2_: C, 30.41; H, 2.19; N, 20.26. Found: C, 30.51; H, 2.55; N, 19.78. Λ_M_ (S cm^2^ mol^−1^): 162.44, within the typical range for a 1:2 electrolyte in water, 150–310 S cm^2^ mol^−1^ (Geary, 1971 ▸). ESI–MS (methanol): *m*/*z* 270.03 [*M* ^2+^]. FT–IR (cm^−1^): 559, 708, 796, 844, 1102, 1409, 1629, 1590, 1551, 1471. Crystals of the title compound were obtained by slow evaporation of a methanol:water solution of the complex.

## Refinement   

Crystal data, data collection and structure refinement details are summarized in Table 2 ▸. Hydrogen atoms bonded to carbon and nitro­gen atoms were added to the structure in idealized positions (N—H = 0.88, C—H = 0.95 Å) and further refined according to the riding model with *U*_iso_(H) = 1.2*U*_eq_(C,N). During the refinement process, electron densities near oxygen atoms were not found in difference maps, resulting in missing hydrogen atoms for water mol­ecules. This is probably a consequence of disordered hydrogen positions resulting from weak inter­molecular inter­actions between lattice water mol­ecules and anions in the structure. The crystal was a strong absorber and exposure times had to be increased in order to achieve a reasonable completeness. In the end, we tested three different absorption correction methods in order to avoid artefacts and the multi-scan method gave the best results. However, a residual positive density was still found close to ruthenium (less than 1 Å) as a consequence of this insufficient absorption correction (Spek, 2018 ▸).

## Supplementary Material

Crystal structure: contains datablock(s) I. DOI: 10.1107/S2056989018007995/wm5447sup1.cif

Structure factors: contains datablock(s) I. DOI: 10.1107/S2056989018007995/wm5447Isup2.hkl

Supporting information file. DOI: 10.1107/S2056989018007995/wm5447Isup3.mol

Detailed information on instrumentation, ESI-MS results, UV-Vis and FT-IR spectra, and cyclic voltammetry.. DOI: 10.1107/S2056989018007995/wm5447sup4.pdf

CCDC reference: 1842596

Additional supporting information:  crystallographic information; 3D view; checkCIF report

## Figures and Tables

**Table 1:** Hydrogen-bond geometry (Å, °)

*D*—H⋯*A*	*D*—H	H⋯*A*	*D*⋯*A*	*D*—H⋯*A*
N2—H2⋯N4^i^	0.88	2.13	2.935 (7)	152
N6—H6⋯O3^ii^	0.88	2.00	2.871 (10)	171
N10—H10⋯O3^iii^	0.88	1.94	2.809 (10)	167
O1⋯F9			3.198 (11)	
O1⋯F12^iv^			2.793 (7)	
O2⋯O1^v^			2.703 (7)	
O2⋯F3^vi^			2.895 (10)	
O3⋯O2			2.784 (9)	
O3⋯F11^vii^			2.909 (8)	

Symmetry codes: (i) 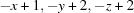; (ii) 
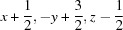
; (iii) 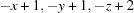; (iv) 
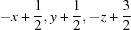
; (v) 
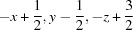
; (vi) 
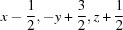
; (vii) 
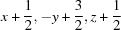
.

**Table 2:** Experimental details

Crystal data	
Chemical formula	[Ru(C_7_H_6_N_4_)_3_](PF_6_)_2_·3H_2_O
*M*_r_	883.49
Crystal system, space group	Monoclinic, *P*2_1_/*n*
Temperature (K)	150
*a*, *b*, *c* (Å)	13.0162 (5), 13.6078 (5), 18.3382 (7)
β (°)	99.937 (2)
*V* (Å^3^)	3199.4 (2)
*Z*	4
Radiation type	Cu *K*α
μ (mm^−1^)	6.02
Crystal size (mm)	0.10 × 0.07 × 0.07

Data collection	
Diffractometer	Bruker APEX CCD detector
Absorption correction	Multi-scan (*SADABS*; Bruker, 2010 ▸)
*T*_min_, *T*_max_	0.625, 0.753
No. of measured, independent and observed [*I* > 2σ(*I*)] reflections	25003, 5754, 4930
*R*_int_	0.039
(sin θ/λ)_max_ (Å^−1^)	0.605

Refinement	
*R*[*F* ^2^ > 2σ(*F* ^2^)], *wR*(*F* ^2^), *S*	0.079, 0.229, 1.08
No. of reflections	5754
No. of parameters	460
H-atom treatment	H-atom parameters constrained
Δρ_max_, Δρ_min_ (e Å^−3^)	3.62, −0.58

Computer programs: *APEX2* and *SAINT* (Bruker, 2010 ▸), *SHELXT* (Sheldrick, 2015*a* ▸), *SHELXL2016* (Sheldrick, 2015*b* ▸) and *OLEX2* (Dolomanov *et al.*, 2009 ▸).

## supplementary crystallographic information

### Crystal data

**Table:**

[Ru(C_7_H_6_N_4_)_3_](PF_6_)_2_·3H_2_O	*F*(000) = 1736
*M**_r_* = 883.49	*D*_x_ = 1.822 Mg m^−^^3^
Monoclinic, *P*2_1_/*n*	Cu *K*α radiation, λ = 1.54178 Å
*a* = 13.0162 (5) Å	Cell parameters from 9950 reflections
*b* = 13.6078 (5) Å	θ = 3.9–68.3°
*c* = 18.3382 (7) Å	µ = 6.02 mm^−^^1^
β = 99.937 (2)°	*T* = 150 K
*V* = 3199.4 (2) Å^3^	Irregular, orange
*Z* = 4	0.10 × 0.07 × 0.07 mm

### Data collection

**Table:**

Bruker APEX CCD detector diffractometer	5754 independent reflections
Radiation source: fine-focus sealed tube	4930 reflections with *I* > 2σ(*I*)
Detector resolution: 8.3333 pixels mm^-1^	*R*_int_ = 0.039
φ and ω scans	θ_max_ = 68.9°, θ_min_ = 3.9°
Absorption correction: multi-scan (SADABS; Bruker, 2010)	*h* = −14→15
*T*_min_ = 0.625, *T*_max_ = 0.753	*k* = −14→16
25003 measured reflections	*l* = −20→22

### Refinement

**Table:**

Refinement on *F*^2^	Primary atom site location: dual
Least-squares matrix: full	Hydrogen site location: inferred from neighbouring sites
*R*[*F*^2^ > 2σ(*F*^2^)] = 0.079	H-atom parameters constrained
*wR*(*F*^2^) = 0.229	*w* = 1/[σ^2^(*F*_o_^2^) + (0.1495*P*)^2^ + 5.4202*P*] where *P* = (*F*_o_^2^ + 2*F*_c_^2^)/3
*S* = 1.08	(Δ/σ)_max_ = 0.001
5754 reflections	Δρ_max_ = 3.62 e Å^−^^3^
460 parameters	Δρ_min_ = −0.58 e Å^−^^3^
0 restraints	

### Special details

**Table:**

Geometry. All esds (except the esd in the dihedral angle between two l.s. planes) are estimated using the full covariance matrix. The cell esds are taken into account individually in the estimation of esds in distances, angles and torsion angles; correlations between esds in cell parameters are only used when they are defined by crystal symmetry. An approximate (isotropic) treatment of cell esds is used for estimating esds involving l.s. planes.

### Fractional atomic coordinates and isotropic or equivalent isotropic displacement parameters (Å^2^)

**Table:**

	*x*	*y*	*z*	*U*_iso_*/*U*_eq_	
Ru1	0.61116 (3)	0.76501 (4)	0.79791 (3)	0.0579 (2)	
N1	0.4844 (4)	0.8111 (4)	0.8410 (3)	0.0657 (13)	
N2	0.4208 (4)	0.9042 (5)	0.9198 (3)	0.0718 (15)	
H2	0.416775	0.948958	0.953833	0.086*	
N3	0.6712 (4)	0.8865 (4)	0.8591 (3)	0.0566 (11)	
N4	0.6301 (4)	1.0022 (4)	0.9467 (3)	0.0582 (12)	
N5	0.7422 (4)	0.7349 (4)	0.7524 (3)	0.0615 (13)	
N6	0.8342 (5)	0.7645 (4)	0.6655 (4)	0.0724 (15)	
H6	0.854183	0.790086	0.626150	0.087*	
N7	0.5863 (4)	0.8536 (4)	0.7038 (3)	0.0602 (12)	
N8	0.6760 (5)	0.9208 (4)	0.6123 (3)	0.0756 (15)	
N9	0.6439 (5)	0.6722 (4)	0.8869 (3)	0.0681 (13)	
N10	0.6215 (6)	0.5297 (5)	0.9368 (4)	0.0879 (19)	
H10	0.600308	0.469110	0.942001	0.105*	
N11	0.5437 (4)	0.6343 (4)	0.7531 (3)	0.0603 (12)	
N12	0.5156 (5)	0.4675 (4)	0.7859 (4)	0.0782 (16)	
C1	0.5046 (5)	0.8819 (5)	0.8901 (3)	0.0623 (15)	
C2	0.3815 (6)	0.7869 (7)	0.8375 (5)	0.081 (2)	
H2A	0.344267	0.738678	0.806039	0.098*	
C3	0.3421 (6)	0.8436 (7)	0.8866 (5)	0.086 (2)	
H3	0.272783	0.841756	0.896368	0.103*	
C4	0.6070 (4)	0.9271 (5)	0.9011 (3)	0.0564 (13)	
C5	0.7256 (5)	1.0409 (5)	0.9491 (3)	0.0619 (14)	
H5	0.744892	1.096557	0.979591	0.074*	
C6	0.7954 (5)	1.0035 (5)	0.9096 (4)	0.0644 (15)	
H6A	0.862998	1.031426	0.913131	0.077*	
C7	0.7666 (5)	0.9254 (5)	0.8651 (4)	0.0617 (14)	
H7	0.814977	0.897782	0.837543	0.074*	
C8	0.7483 (5)	0.7886 (5)	0.6927 (4)	0.0604 (14)	
C9	0.8261 (5)	0.6748 (5)	0.7632 (4)	0.0686 (16)	
H9	0.841453	0.627585	0.801761	0.082*	
C10	0.8851 (5)	0.6927 (6)	0.7102 (4)	0.0749 (19)	
H10A	0.948787	0.661564	0.705115	0.090*	
C11	0.6671 (5)	0.8592 (5)	0.6664 (4)	0.0648 (15)	
C12	0.5938 (8)	0.9800 (6)	0.5917 (5)	0.086 (2)	
H12	0.595776	1.025988	0.553000	0.103*	
C13	0.5090 (7)	0.9766 (6)	0.6239 (5)	0.086 (2)	
H13	0.451247	1.018106	0.606409	0.103*	
C14	0.5047 (6)	0.9149 (6)	0.6809 (5)	0.0757 (19)	
H14	0.445435	0.914399	0.704742	0.091*	
C15	0.6043 (6)	0.5838 (5)	0.8740 (4)	0.0726 (17)	
C16	0.6922 (7)	0.6751 (6)	0.9600 (4)	0.080 (2)	
H16	0.729082	0.729431	0.984397	0.097*	
C17	0.6779 (8)	0.5869 (7)	0.9907 (5)	0.092 (2)	
H17	0.702521	0.568118	1.040599	0.111*	
C18	0.5512 (5)	0.5592 (5)	0.8004 (4)	0.0680 (16)	
C19	0.4696 (6)	0.4537 (6)	0.7149 (5)	0.082 (2)	
H19	0.443343	0.390098	0.700580	0.099*	
C20	0.4588 (5)	0.5240 (5)	0.6637 (4)	0.0718 (18)	
H20	0.425902	0.510496	0.614305	0.086*	
C21	0.4959 (5)	0.6162 (6)	0.6833 (4)	0.0662 (16)	
H21	0.487895	0.667494	0.647555	0.079*	
P1	0.52669 (15)	0.72394 (12)	0.47516 (11)	0.0672 (5)	
F1	0.6051 (4)	0.6755 (5)	0.4292 (3)	0.1094 (18)	
F2	0.4541 (6)	0.7727 (7)	0.5259 (5)	0.143 (3)	
F3	0.5632 (6)	0.8307 (4)	0.4586 (3)	0.120 (2)	
F4	0.4894 (5)	0.6168 (4)	0.4927 (3)	0.117 (2)	
F5	0.4400 (5)	0.7282 (4)	0.4045 (4)	0.119 (2)	
F6	0.6122 (4)	0.7171 (3)	0.5476 (3)	0.0905 (14)	
P2	0.17757 (17)	0.6704 (2)	0.65300 (15)	0.0922 (7)	
F7	0.2160 (5)	0.5967 (8)	0.5979 (4)	0.166 (4)	
F8	0.1418 (6)	0.7402 (6)	0.7124 (7)	0.161 (4)	
F9	0.2594 (7)	0.7480 (6)	0.6365 (6)	0.150 (3)	
F10	0.0961 (5)	0.5899 (5)	0.6745 (4)	0.129 (2)	
F11	0.0909 (7)	0.7042 (13)	0.5912 (8)	0.233 (6)	
F12	0.2637 (5)	0.6267 (5)	0.7163 (4)	0.1182 (19)	
O1	0.2542 (8)	0.9735 (9)	0.6848 (6)	0.165 (4)	
O2	0.2613 (8)	0.6190 (7)	0.9169 (7)	0.153 (3)	
O3	0.4135 (6)	0.6699 (5)	1.0371 (4)	0.112 (2)	

### Atomic displacement parameters (Å^2^)

**Table:**

	*U*^11^	*U*^22^	*U*^33^	*U*^12^	*U*^13^	*U*^23^
Ru1	0.0485 (3)	0.0616 (4)	0.0631 (3)	−0.00170 (17)	0.0080 (2)	−0.01872 (19)
N1	0.056 (3)	0.069 (3)	0.073 (3)	−0.013 (2)	0.014 (2)	−0.027 (3)
N2	0.054 (3)	0.081 (4)	0.083 (3)	−0.014 (3)	0.019 (3)	−0.037 (3)
N3	0.049 (2)	0.059 (3)	0.060 (3)	0.001 (2)	0.005 (2)	−0.013 (2)
N4	0.053 (3)	0.062 (3)	0.060 (3)	−0.005 (2)	0.010 (2)	−0.015 (2)
N5	0.051 (3)	0.069 (3)	0.062 (3)	0.004 (2)	0.005 (2)	−0.018 (2)
N6	0.063 (3)	0.078 (4)	0.081 (4)	−0.006 (3)	0.024 (3)	−0.016 (3)
N7	0.059 (3)	0.053 (3)	0.066 (3)	0.003 (2)	0.003 (2)	−0.014 (2)
N8	0.085 (4)	0.059 (3)	0.080 (4)	−0.011 (3)	0.008 (3)	−0.009 (3)
N9	0.068 (3)	0.072 (3)	0.064 (3)	−0.007 (3)	0.011 (2)	−0.009 (3)
N10	0.101 (5)	0.068 (4)	0.096 (4)	−0.005 (3)	0.022 (4)	−0.007 (3)
N11	0.052 (3)	0.060 (3)	0.071 (3)	0.002 (2)	0.016 (2)	−0.014 (2)
N12	0.082 (4)	0.062 (3)	0.091 (4)	−0.002 (3)	0.016 (3)	−0.016 (3)
C1	0.050 (3)	0.072 (4)	0.066 (3)	−0.006 (3)	0.013 (3)	−0.020 (3)
C2	0.060 (4)	0.093 (5)	0.093 (5)	−0.027 (4)	0.018 (4)	−0.039 (4)
C3	0.058 (4)	0.106 (6)	0.100 (5)	−0.020 (4)	0.029 (4)	−0.047 (5)
C4	0.045 (3)	0.062 (3)	0.061 (3)	−0.001 (2)	0.006 (2)	−0.013 (3)
C5	0.059 (3)	0.064 (3)	0.062 (3)	−0.011 (3)	0.009 (3)	−0.012 (3)
C6	0.051 (3)	0.069 (4)	0.073 (4)	−0.008 (3)	0.010 (3)	−0.008 (3)
C7	0.046 (3)	0.068 (4)	0.070 (3)	−0.001 (3)	0.008 (3)	−0.009 (3)
C8	0.055 (3)	0.060 (3)	0.065 (3)	−0.001 (3)	0.007 (3)	−0.016 (3)
C9	0.056 (3)	0.072 (4)	0.076 (4)	0.008 (3)	0.007 (3)	−0.015 (3)
C10	0.053 (4)	0.080 (5)	0.091 (5)	0.007 (3)	0.010 (3)	−0.018 (4)
C11	0.062 (3)	0.060 (3)	0.071 (4)	−0.008 (3)	0.005 (3)	−0.019 (3)
C12	0.102 (6)	0.064 (4)	0.084 (5)	0.001 (4)	−0.002 (4)	−0.005 (4)
C13	0.087 (5)	0.067 (4)	0.096 (5)	0.009 (4)	−0.003 (4)	−0.001 (4)
C14	0.063 (4)	0.069 (4)	0.090 (5)	0.009 (3)	0.001 (3)	−0.020 (4)
C15	0.076 (4)	0.065 (4)	0.077 (4)	−0.001 (3)	0.015 (3)	−0.002 (3)
C16	0.088 (5)	0.084 (5)	0.068 (4)	−0.017 (4)	0.010 (4)	−0.009 (4)
C17	0.110 (6)	0.094 (6)	0.071 (4)	−0.003 (5)	0.010 (4)	−0.012 (4)
C18	0.059 (3)	0.065 (4)	0.083 (4)	−0.004 (3)	0.018 (3)	−0.026 (3)
C19	0.077 (4)	0.072 (4)	0.099 (5)	−0.012 (4)	0.017 (4)	−0.033 (4)
C20	0.060 (4)	0.069 (4)	0.086 (4)	−0.006 (3)	0.013 (3)	−0.027 (4)
C21	0.052 (3)	0.075 (4)	0.071 (4)	0.004 (3)	0.012 (3)	−0.023 (3)
P1	0.0650 (10)	0.0576 (9)	0.0735 (10)	0.0126 (7)	−0.0032 (8)	−0.0036 (7)
F1	0.093 (3)	0.132 (5)	0.101 (3)	0.020 (3)	0.011 (3)	−0.038 (3)
F2	0.123 (5)	0.171 (7)	0.137 (6)	0.051 (5)	0.030 (5)	−0.020 (5)
F3	0.166 (6)	0.067 (3)	0.117 (4)	−0.012 (3)	−0.005 (4)	0.010 (3)
F4	0.128 (4)	0.083 (3)	0.122 (4)	−0.031 (3)	−0.030 (3)	0.013 (3)
F5	0.109 (4)	0.105 (4)	0.121 (4)	0.005 (3)	−0.046 (4)	0.020 (3)
F6	0.108 (3)	0.064 (2)	0.085 (3)	0.007 (2)	−0.022 (3)	−0.014 (2)
P2	0.0638 (11)	0.1057 (16)	0.1053 (15)	0.0035 (11)	0.0099 (10)	−0.0108 (13)
F7	0.097 (4)	0.252 (10)	0.153 (6)	−0.040 (5)	0.036 (4)	−0.118 (7)
F8	0.099 (5)	0.140 (6)	0.254 (11)	0.001 (4)	0.054 (6)	−0.085 (6)
F9	0.121 (6)	0.168 (7)	0.164 (7)	−0.038 (5)	0.037 (5)	0.003 (5)
F10	0.087 (3)	0.122 (4)	0.186 (6)	−0.016 (3)	0.047 (4)	−0.048 (4)
F11	0.094 (5)	0.350 (16)	0.234 (12)	−0.002 (8)	−0.029 (6)	0.112 (12)
F12	0.093 (3)	0.135 (5)	0.122 (4)	0.011 (3)	0.005 (3)	−0.025 (4)
O1	0.146 (8)	0.188 (11)	0.163 (8)	−0.016 (7)	0.032 (7)	−0.032 (8)
O2	0.148 (8)	0.113 (6)	0.198 (9)	−0.018 (6)	0.028 (7)	0.019 (6)
O3	0.129 (5)	0.081 (4)	0.139 (5)	−0.006 (4)	0.058 (5)	0.002 (4)

### Geometric parameters (Å, º)

**Table:**

Ru1—N1	2.047 (5)	C3—H3	0.9500
Ru1—N3	2.074 (5)	C5—H5	0.9500
Ru1—N5	2.066 (6)	C5—C6	1.355 (9)
Ru1—N7	2.084 (5)	C6—H6A	0.9500
Ru1—N9	2.050 (6)	C6—C7	1.353 (9)
Ru1—N11	2.089 (5)	C7—H7	0.9500
N1—C1	1.314 (8)	C8—C11	1.449 (9)
N1—C2	1.370 (9)	C9—H9	0.9500
N2—H2	0.8800	C9—C10	1.362 (11)
N2—C1	1.335 (8)	C10—H10A	0.9500
N2—C3	1.373 (9)	C12—H12	0.9500
N3—C4	1.349 (8)	C12—C13	1.339 (13)
N3—C7	1.337 (8)	C13—H13	0.9500
N4—C4	1.322 (8)	C13—C14	1.350 (12)
N4—C5	1.344 (8)	C14—H14	0.9500
N5—C8	1.330 (9)	C15—C18	1.446 (10)
N5—C9	1.351 (9)	C16—H16	0.9500
N6—H6	0.8800	C16—C17	1.353 (13)
N6—C8	1.341 (9)	C17—H17	0.9500
N6—C10	1.370 (11)	C19—H19	0.9500
N7—C11	1.352 (9)	C19—C20	1.331 (12)
N7—C14	1.359 (9)	C20—H20	0.9500
N8—C11	1.320 (9)	C20—C21	1.370 (10)
N8—C12	1.341 (11)	C21—H21	0.9500
N9—C15	1.314 (10)	P1—F1	1.576 (5)
N9—C16	1.379 (9)	P1—F2	1.582 (7)
N10—H10	0.8800	P1—F3	1.574 (6)
N10—C15	1.352 (10)	P1—F4	1.587 (6)
N10—C17	1.369 (11)	P1—F5	1.565 (5)
N11—C18	1.333 (10)	P1—F6	1.581 (5)
N11—C21	1.346 (9)	P2—F7	1.565 (7)
N12—C18	1.342 (9)	P2—F8	1.575 (8)
N12—C19	1.349 (10)	P2—F9	1.566 (8)
C1—C4	1.449 (8)	P2—F10	1.619 (7)
C2—H2A	0.9500	P2—F11	1.526 (9)
C2—C3	1.352 (11)	P2—F12	1.584 (7)
			
N1—Ru1—N3	78.47 (19)	N5—C9—H9	125.5
N1—Ru1—N5	173.6 (2)	N5—C9—C10	109.1 (7)
N1—Ru1—N7	97.0 (2)	C10—C9—H9	125.5
N1—Ru1—N9	87.2 (2)	N6—C10—H10A	126.8
N1—Ru1—N11	95.8 (2)	C9—C10—N6	106.3 (6)
N3—Ru1—N7	88.69 (19)	C9—C10—H10A	126.8
N3—Ru1—N11	170.3 (2)	N7—C11—C8	112.4 (6)
N5—Ru1—N3	96.7 (2)	N8—C11—N7	126.5 (6)
N5—Ru1—N7	78.5 (2)	N8—C11—C8	121.1 (7)
N5—Ru1—N11	89.6 (2)	N8—C12—H12	118.9
N7—Ru1—N11	99.9 (2)	C13—C12—N8	122.3 (8)
N9—Ru1—N3	93.1 (2)	C13—C12—H12	118.9
N9—Ru1—N5	97.3 (2)	C12—C13—H13	119.7
N9—Ru1—N7	175.6 (2)	C12—C13—C14	120.7 (8)
N9—Ru1—N11	78.7 (2)	C14—C13—H13	119.7
C1—N1—Ru1	114.2 (4)	N7—C14—H14	120.4
C1—N1—C2	106.7 (6)	C13—C14—N7	119.1 (8)
C2—N1—Ru1	139.0 (5)	C13—C14—H14	120.4
C1—N2—H2	126.8	N9—C15—N10	110.0 (7)
C1—N2—C3	106.4 (5)	N9—C15—C18	119.3 (7)
C3—N2—H2	126.8	N10—C15—C18	130.6 (7)
C4—N3—Ru1	115.0 (4)	N9—C16—H16	126.1
C7—N3—Ru1	128.2 (4)	C17—C16—N9	107.9 (7)
C7—N3—C4	116.7 (5)	C17—C16—H16	126.1
C4—N4—C5	115.5 (5)	N10—C17—H17	126.3
C8—N5—Ru1	113.2 (4)	C16—C17—N10	107.4 (8)
C8—N5—C9	107.0 (6)	C16—C17—H17	126.3
C9—N5—Ru1	139.7 (5)	N11—C18—N12	126.9 (6)
C8—N6—H6	126.3	N11—C18—C15	113.5 (6)
C8—N6—C10	107.4 (6)	N12—C18—C15	119.6 (7)
C10—N6—H6	126.3	N12—C19—H19	118.1
C11—N7—Ru1	115.4 (4)	C20—C19—N12	123.8 (7)
C11—N7—C14	116.3 (6)	C20—C19—H19	118.1
C14—N7—Ru1	127.8 (5)	C19—C20—H20	120.5
C11—N8—C12	115.1 (7)	C19—C20—C21	119.0 (7)
C15—N9—Ru1	113.5 (5)	C21—C20—H20	120.5
C15—N9—C16	107.5 (7)	N11—C21—C20	120.0 (7)
C16—N9—Ru1	138.9 (5)	N11—C21—H21	120.0
C15—N10—H10	126.4	C20—C21—H21	120.0
C15—N10—C17	107.2 (7)	F1—P1—F2	176.2 (4)
C17—N10—H10	126.4	F1—P1—F4	88.4 (4)
C18—N11—Ru1	114.7 (4)	F1—P1—F6	89.8 (3)
C18—N11—C21	116.7 (6)	F2—P1—F4	91.8 (5)
C21—N11—Ru1	128.6 (5)	F3—P1—F1	92.2 (4)
C18—N12—C19	113.7 (7)	F3—P1—F2	87.5 (5)
N1—C1—N2	111.4 (5)	F3—P1—F4	179.3 (4)
N1—C1—C4	118.5 (5)	F3—P1—F6	91.4 (3)
N2—C1—C4	130.0 (5)	F5—P1—F1	90.8 (4)
N1—C2—H2A	125.9	F5—P1—F2	93.0 (4)
C3—C2—N1	108.2 (6)	F5—P1—F3	90.3 (3)
C3—C2—H2A	125.9	F5—P1—F4	90.0 (3)
N2—C3—H3	126.3	F5—P1—F6	178.2 (4)
C2—C3—N2	107.4 (6)	F6—P1—F2	86.4 (4)
C2—C3—H3	126.3	F6—P1—F4	88.3 (3)
N3—C4—C1	113.1 (5)	F7—P2—F8	176.5 (6)
N4—C4—N3	125.6 (5)	F7—P2—F9	90.2 (5)
N4—C4—C1	121.2 (5)	F7—P2—F10	91.1 (4)
N4—C5—H5	118.7	F7—P2—F12	88.2 (4)
N4—C5—C6	122.6 (6)	F8—P2—F10	87.4 (4)
C6—C5—H5	118.7	F8—P2—F12	88.6 (5)
C5—C6—H6A	120.8	F9—P2—F8	91.1 (5)
C7—C6—C5	118.4 (6)	F9—P2—F10	177.0 (5)
C7—C6—H6A	120.8	F9—P2—F12	88.6 (5)
N3—C7—C6	121.2 (6)	F11—P2—F7	89.5 (8)
N3—C7—H7	119.4	F11—P2—F8	93.6 (8)
C6—C7—H7	119.4	F11—P2—F9	95.4 (6)
N5—C8—N6	110.1 (6)	F11—P2—F10	87.4 (6)
N5—C8—C11	119.6 (6)	F11—P2—F12	175.4 (7)
N6—C8—C11	130.2 (7)	F12—P2—F10	88.7 (4)

### Hydrogen-bond geometry (Å, º)

**Table:**

*D*—H···*A*	*D*—H	H···*A*	*D*···*A*	*D*—H···*A*
N2—H2···N4^i^	0.88	2.13	2.935 (7)	152
N6—H6···O3^ii^	0.88	2.00	2.871 (10)	171
N10—H10···O3^iii^	0.88	1.94	2.809 (10)	167
O1···F9			3.198 (11)	
O1···F12^iv^			2.793 (7)	
O2···O1^v^			2.703 (7)	
O2···F3^vi^			2.895 (10)	
O3···O2			2.784 (9)	
O3···F11^vii^			2.909 (8)	

Symmetry codes: (i) −*x*+1, −*y*+2, −*z*+2; (ii) *x*+1/2, −*y*+3/2, *z*−1/2; (iii) −*x*+1, −*y*+1, −*z*+2; (iv) −*x*+1/2, *y*+1/2, −*z*+3/2; (v) −*x*+1/2, *y*−1/2, −*z*+3/2; (vi) *x*−1/2, −*y*+3/2, *z*+1/2; (vii) *x*+1/2, −*y*+3/2, *z*+1/2.
