# Supplementary material for: Crystal structure, electrochemical and spectroscopic investigation of mer-tris­[2-(1H-imidazol-2-yl-κN 3)pyrimidine-κN 1]ruthenium(II) bis­(hexa­fluorido­phosphate) trihydrate
Source: Acta Crystallogr E Crystallogr Commun. 2018 Jun 5;74(Pt 7):874–7. doi: 10.1107/S2056989018007995 (PMC6038643; doi:10.1107/S2056989018007995)
Supplement: Supplementary file 4 [file e-74-00874-sup4.pdf]

Crystal structure, electrochemical and spectroscopic investigation of *mer*-tris[2-(1*H*-imidazol-2-yl-κ*N*[3])pyrimidine-κ[21]] ruthenium(II) bis(hexafluoridophosphate) trihydrate

Naheed Bibi, Renan Barrach Guerra, Luis Enrique Santa Cruz Huamaní and André Luiz Barboza Formiga*

Institute of Chemistry, University of Campinas – UNICAMP, PO Box 6154, 13083-970, Campinas, SP, Brazil

Correspondence email: formiga@g.unicamp.br

doi: 10.1107/S2056989018007995

Supporting Information

Physical measurements

Elemental analyses of the complex were performed using a Perkin Elmer 2400 CHN analyser.

Electrospray ionization mass spectra (ESI-MS) were recorded with a Waters Quattro Micro API in a positive mode using a 1:1 methanol:water solution with addition of 0.10% (v/v) formic acid. Electronic absorption spectra of the complex were obtained using a quartz cuvette (1.00 cm) with a diode array HP8453 UV/Visible absorption spectrophotometer equipped with a HP89090A Peltier.

Infrared spectra were obtained with an Agilent Cary 630 FTIR spectrometer, using the Attenuated Total Reflectance (ATR) method, with a diamond cell. The spectra were recorded in 4000-400 cm^{-1} range, with 64 scans and a resolution of 4 cm^{-1}.

For cyclic voltammetry, vitreous carbon, platinum and Ag/AgCl were employed as working, counter and a reference electrode, respectively. Solutions of the complexes ($5x10^{-3}$ mol L^{-1}) were prepared in a pH 1 buffer solution (25 ml of 0.2 M KCl + 67 ml of 0.2 M HNO_3). The working electrode was cleaned and polished with fine alumina slurries of various granular sizes on a microcloth pad for effective removal of any previously adsorbed species. A blank was run with the buffer solution, followed by various cyclic voltammograms in different ranges with scan rates of 10, 25, 50, 100 and 200 mV s^{-1}.

For conductivity measurements, LAB1000 conductometer and a standard platinum electrode cell with a cell constant of 0.1 was used. The measurements were acquired using $9.8x10^{-4}$ mol L^{-1} aqueous solutions of the complex.

Mass Spectrometry

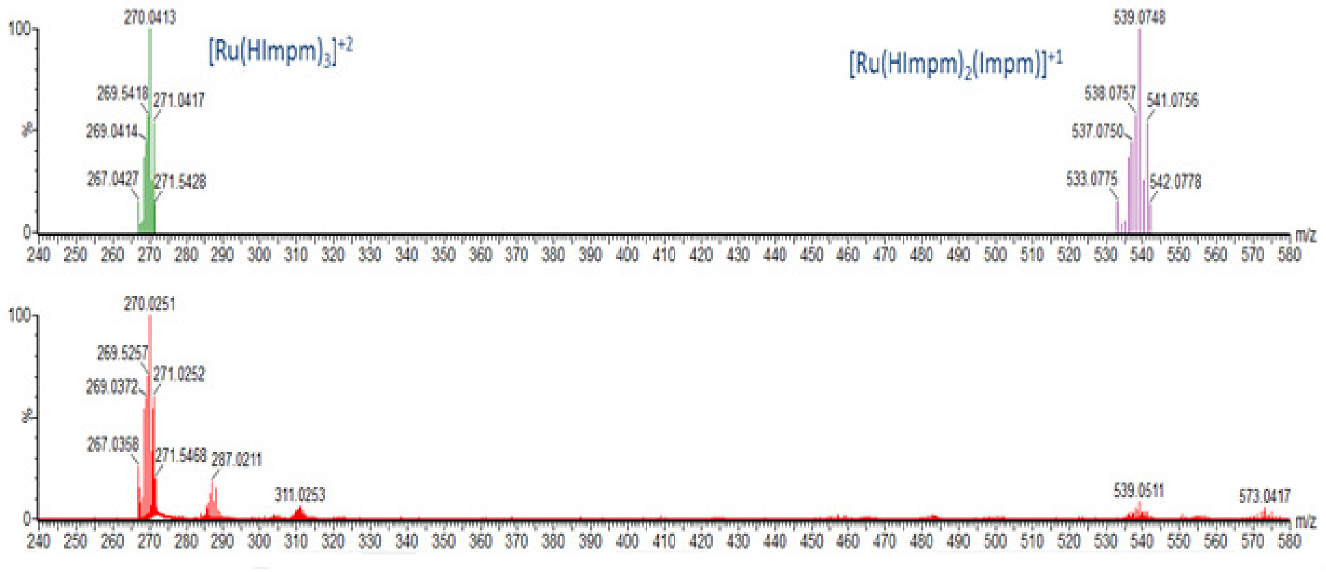

Figure S1. Simulated (above) and experimental (below) high resolution ESI-MS spectra of the complex [Ru(Himpm)$_3$](PF$_6$)$_2$ in methanol, obtained in positive mode.

Electronic Absorption Spectroscopy

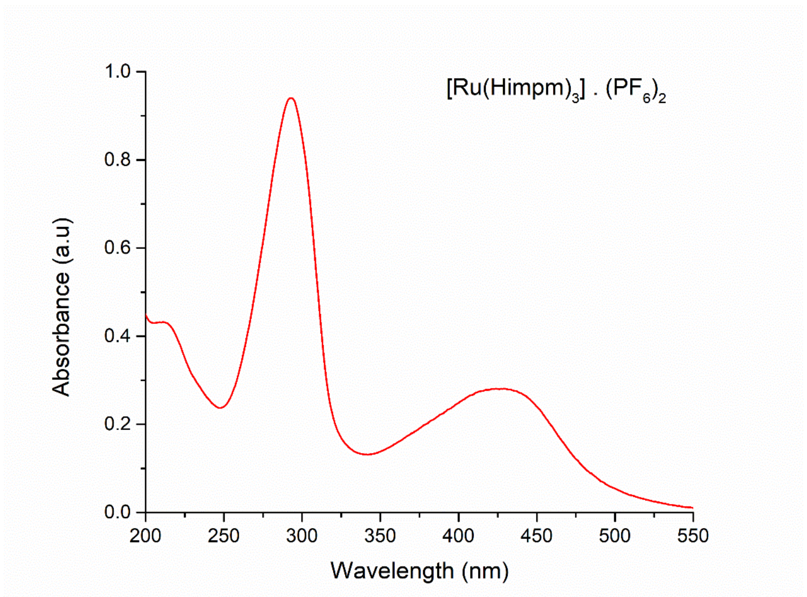

Figure S2. Electronic absorption spectrum of [Ru(Himpm)$_3$](PF$_6$)$_2$ showing absorptions over the UV-visible range in H_2O (1.9778 x 10^{-5} mol L^{-1}). Maxima: 293 nm (log ε = 4.67), 421 nm (log ε = 4.14).

Infrared Spectroscopy

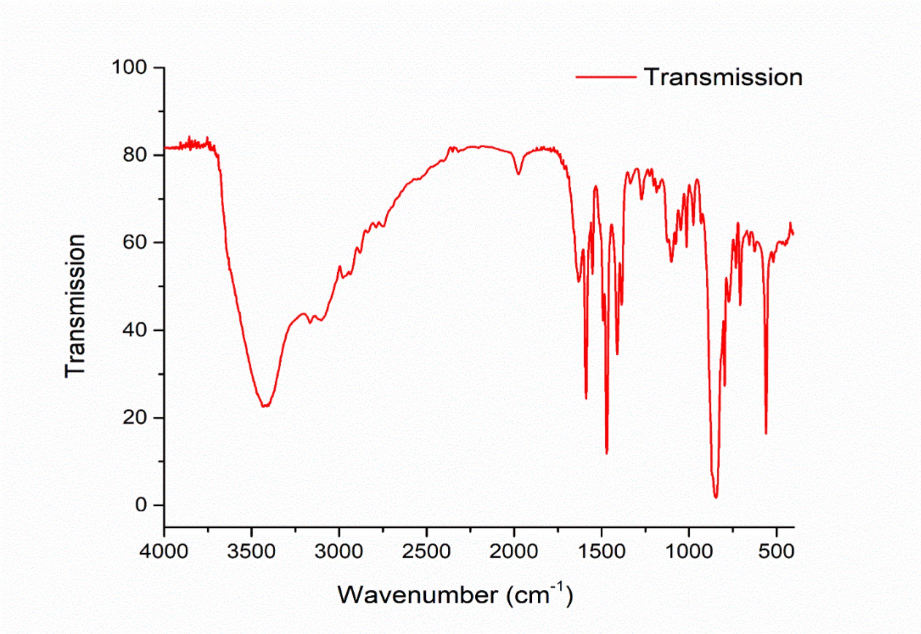

Figure S3. ATR Infrared spectrum of [Ru(Himpm)$_3$](PF$_6$)$_2$

Cyclic Voltammetry

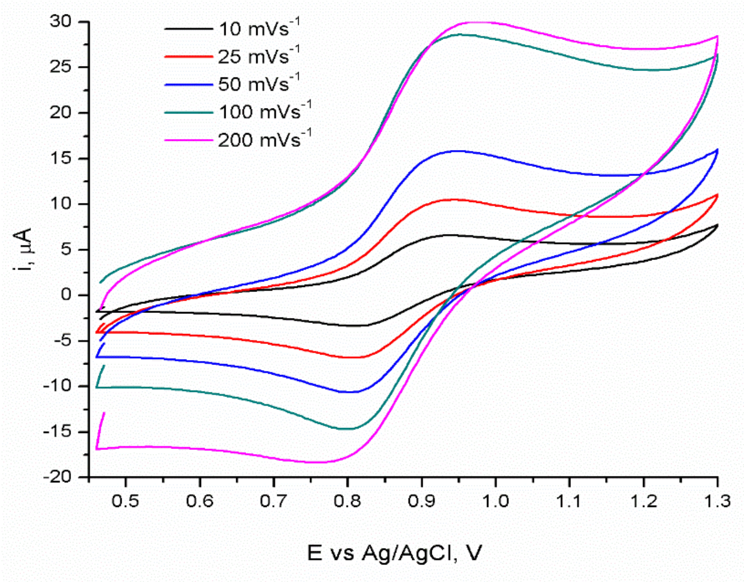

Figure S4. Cyclic voltammogram of [Ru(Himpm)$_3$](PF$_6$)$_2$ ($5x10^{-3}$ mol L^{-1}) in buffer of pH 1 (25 ml of 0.2 M KNO_3 + 67 ml of 0.2 M HNO_3).
